# The SMOC1 proteomics network controls signaling modalities, brain homeostasis and toxicity in fly Alzheimer's disease models

**DOI:** 10.1002/alz70855_103456

**Published:** 2025-12-23

**Authors:** David Li‐Kroeger, Ismael Al‐Ramahi, Nathaniel Smith, Aditi Marella, Bismark K Amoh, Saathwik Saladi, Ruhee Marfathia, Juan Botas, Nicholas T Seyfried, Allan I. Levey, Joshua M Shulman

**Affiliations:** ^1^ Baylor College of Medicine ‐ NRI, Houston, TX, USA; ^2^ Baylor College of Medicine, Houston, TX, USA; ^3^ Jan and Dan Duncan Neurological Research Institute, Texas Children's Hospital, Houston, TX, USA; ^4^ Brandeis Universtiy, Waltham, MA, USA; ^5^ Des Moines University, Des Moines, IA, USA; ^6^ University of California, Los Angeles, CA, USA; ^7^ Rice University, houston, TX, USA; ^8^ Emory University School of Medicine, Atlanta, GA, USA

## Abstract

**Background:**

Alzheimer's disease (AD) has a complex etiology where insults in multiple pathways collude to disrupt neuronal function, yet the molecular changes underlying AD remain poorly understood. To identify changes associated with AD, we previously performed mass‐spectrometry on post‐mortem brain tissue and identified protein co‐expression networks correlated to AD pathological and clinical traits. The network most strongly correlated with AD pathology is enriched in components of the matrisome and includes multiple signaling pathways, Aß, APOE, and SMOC1, a predicted driver of network behavior. SMOC1 is a matrisomal protein with conserved roles in modulating signaling during development, yet its role in the adult brain remains unstudied.

**Methods:**

We evaluate the SMOC1 protein network using the powerful genetics of *Drosophila melanogaster* to determine its role in the brain and uncover links to specific triggers of AD: Amyloid‐Beta (Aβ) and tau. We use a high‐throughput robotic screening platform and video assisted software enabling quantitative assessments of neurological function to identify proteins modifying Aβ‐ or tau‐induced neurodegeneration. We use Mass‐Spectrometry to identify protein changes in the brains of flies with loss‐of‐function mutations in dSMOC1 and genetics, cell biology, and immunofluorescence to characterize dSMOC1 function.

**Results:**

We identified 25 genes from the SMOC1 network with robust evidence of interactions with either tau or Aβ toxicity in fly AD models. Consistent with its central, interconnected position within the network, dSMOC1 shows genetic interactions with at least 8 other network genes. *dSMOC1* is expressed in the brain by many glia and some neurons, however, dSMOC1 protein is mainly localized around neuronal cell bodies. dSMOC1^‐/‐^ mutant flies have severe locomotor defects, reduced survival, and show cell‐type specific changes in glypican expression, potentially linking the network to Wnt and TGF‐β signaling. Finally, proteomics from dSMOC1^‐/‐^ fly heads show perturbations in ECM/Receptor interactions, metabolism, signaling, and proteostasis

**Conclusion:**

The SMOC1 protein network contains multiple proteins with links to AD, and dSMOC1 is likely a key driver of network function. Together, the genetics and proteomics data show that dSMOC1 is an important regulator of extracellular matrix function in the brain and suggest a mechanistic hypothesis where dSMOC1 fine‐tunes multiple signaling modalities to regulate cellular homeostasis.

